# The arsenal of TP53 mutants therapies: neoantigens and bispecific antibodies

**DOI:** 10.1038/s41392-021-00635-y

**Published:** 2021-06-03

**Authors:** Chang Yang, Ge Lou, Wei-Lin Jin

**Affiliations:** 1 Department of Gynecology Oncology, The Tumor Hospital, Harbin Medical University, Harbin, P.R. China; 2 Institute of Cancer Neuroscience, Medical Frontier Innovation Research Center, The First Hospital of Lanzhou University, The First Clinical Medical College of Lanzhou University, Lanzhou, P.R. China

**Keywords:** Drug development, Target identification

A recent research published in Science by Hsiue et al.^1^ introduced a CD3-targeting bispecific antibody that can bind to tumor cells by recognizing mutation-associated neoantigens and activate T cell-mediated tumor killing by binding to CD3.

**Fig. 1:**
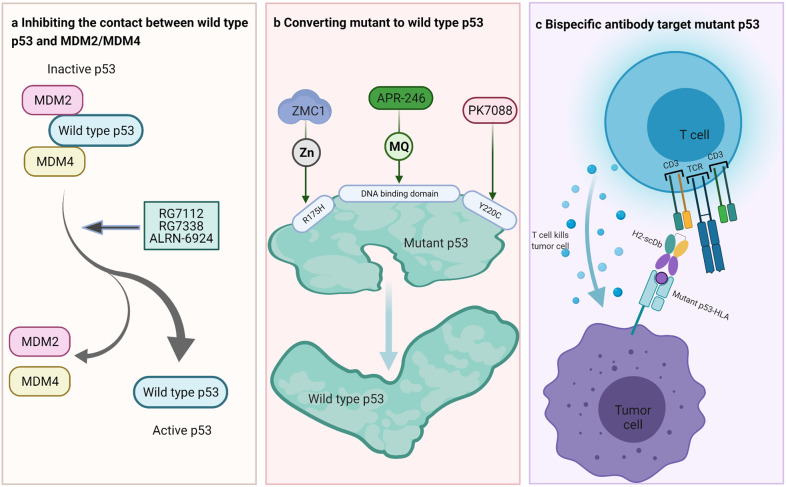
Treatment strategies of p53 dysfunction. **a** Inhibition of the interaction between wild-type p53 and MDM2/MDM4: RG7112 and RG7338 are MDM2 inhibitors that hinder direct interaction between p53 and MDM2. ALRN-6924 is a dual MDM2/MDM4 inhibitor. RG7112, RG7338, and ALRN-6924 are currently undergoing clinical trials. **b** Restoration of wild-type p53 function: zinc metallochaperone-1 (ZMC1) restores the low Zn^2+^ affinity of mutant p53 induced by R175H mutation and enables mutant p53 to fold correctly. APR-246 is converted to methylene quinuclidinone (MQ), a Michael acceptor that reacts with cysteines in the p53 core domain, promoting wild-type p53 conformation. PK7088 binds to Y220C mutant, regulating tail protein stability and enhancing correct protein folding of mutant p53. **c** Bispecific antibody targeting mutant p53: H2-scDb binds to the p53 R175H peptide–HLA complex expressed on the tumor cell surface with one arm and to CD3 with the other arm, inducing T-cell activation and tumor killing

The tumor suppressor gene *TP53* is the most commonly mutated gene in various cancers. More than half of p53 mutations are missense mutations in the core domain, especially in several “mutational hotspots,” including arginine-to-histidine substitution at codon 175 (R175H).^2^ Missense p53 mutation imparts gain-of-function properties, including the inability to control cell proliferation, suppression of apoptosis, and development of chemotherapy resistance,^2^ which result in tumor progression. p53 mutations play essential roles in cancer pathogenesis and are attractive targets for therapy. Current p53-targeting therapeutic strategies are directed at two aspects. For p53 wild-type tumors, the approach is to suppress the interaction between p53 and MDM2/MDM4, block p53 degradation, and maintain p53 density in cells, thus enhancing its capacity to suppress tumorigenesis (Fig. 1a).^2^ For p53 mutant type tumors, therapeutic strategies involve the reactivation of wild-type p53 functions or promotion of mutant p53 degradation (Fig. 1b). Unfortunately, given the “not overactive” nature of mutant p53 and its nuclear location, it is difficult to target, and the development of safe pharmacologic agents to reactivate mutant p53 remains challenging. Decades after the discovery of the critical role of p53 dysfunction in malignancy, therapeutic drugs targeting mutant p53 are unavailable in clinic.

Recently, Hsiue et al. designed a bispecific antibody (BsAb) targeting mutant p53 peptide–human leukocyte antigen (HLA) complex (Fig. 1c).^1^ BsAbs are junctions of two different single-chain variable fragments, one of which is usually directed against neoantigens and the other against CD3, a subunit of the T-cell receptor (TCR) complex, which can activate T cells. Hsiue et al. found that a peptide derived from the p53 R175H missense mutation can bind to a particular HLA allele (HLA-A*02:01) and form a mutant p53 peptide–HLA complex on the cell surface as a natural TCR ligand that can be used to activate T cells. However, the density of the mutant p53 peptide–HLA complex on the cell surface was too low to effectively attract T cells to the cancer cells. To address this problem, Hsiue et al. searched a large phage library and finally found an H2 antibody fragment, whose structure assembled like a cage around the mutant amino acid (His175) and one adjacent residue (Arg174). Owing to this cage-like structure, the H2 antibody fragment had a higher affinity toward the p53 R175H peptide–HLA complex than that toward its wild-type counterpart. The researchers fused the H2 antibody fragment with a CD3 antibody fragment to establish BsAbs that could augment the activation of T cells to enhance the recognition and destruction of cancer cells expressing p53 R175H peptide–HLA complex. In vitro and in vivo experiments demonstrated that the BsAbs targeting the p53 R175H peptide–HLA complex effectively activated T cells and killed tumor cells. These results provided a prospective application for other mutations that are difficult to target by conventional therapeutic approaches. BsAbs have recently been widely used in targeting mutant RAS proteins^3^ and the TCR β chain to treat T-cell malignancies.^4^ The TCR β chain variable gene (TRBV) family comprises TRBV1 to TRBV30. It has been hypothesized that healthy human peripheral blood T cells express multiple TRBV family members on their cell surface, whereas clonal T-cell tumors express only one TRBV. Based on this theory, Paul et al. designed BsAbs targeting TRBV5-5 (α-V5) or TRBV12 (α-V12) that could specifically destruct T-cell malignancies and maintain healthy peripheral blood T cells in vitro and in vivo.^4^

Neoantigen vaccines and adoptive T-cell therapies targeting neo-epitopes have been proven curative in cancer patients. However, the immune escape feature of tumor cells restrains the efficacy of neoantigen vaccines in activating specific T cells. The application of adoptive T-cell therapy is limited by the requirements for patients’ autologous cells and sophisticated manipulation for a personalized approach.^1^ In contrast, BsAbs are easy to manufacture and relatively inexpensive. BsAb-based immunotherapy may shift the treatment landscape and outlook for patients with malignant tumors. Owing to their higher affinity toward mutant neoantigen peptide–HLA complex than to the wild-type counterpart, BsAbs should demonstrate a high specificity for malignant tumor cells. Moreover, although the density of mutant neoantigen peptide–HLA complex expressed on tumor surface is low, fusion with an antibody fragment targeting the CD3 complex on T cells is expected to provide an efficient and powerful signal for T-cell activation. Further, as most HLA haplotype subtypes can bind the same neoantigens (e.g., HLA-A*02:01-02:07), H2-scDb originally designed for targeting p53 R175H peptide–HLA-A*02:01 may be effective in patients with other HLA-A*02 haplotypes, such as HLA-A*02:07, which is common in Hong Kong Chinese. Finally, as p53 mutations are widely prevalent in human cancers, BsAbs targeting mutant p53 peptide–HLA complex may be applicable in multiple types of cancers.

Although the ultimate goal is to apply BsAbs in clinical therapies, there still are theoretical and practical issues with single-chain BsAbs targeting mutant tumor neoantigens. First, single-chain BsAbs are small molecules that are rapidly cleared from the blood in humans and mice and therefore lack a sustained effect.^5^ Second, human classical HLA class I is highly polymorphic, narrowing the range of patients that can be treated with mutation-associated neoantigen peptide–HLA-targeted therapy.^5^ For example, HLA-A*02:01 is one of the most common HLA haplotypes in Caucasians and Native Americans but varies among different ethnicities.

In conclusion, the studies of Hsiue et al. have paved the way to the development of more potent, new strategies targeting mutation-associated neoantigens; however, substantial preclinical work will be required before this approach can be translated into clinical treatments.
